# Sarcoidosis with necrobiosis‐lipoidica–like skin lesions: A challenge for the dermatologist

**DOI:** 10.1002/hsr2.200

**Published:** 2020-12-21

**Authors:** Ingrid Campos, Fernanda Simoneti, Gabriela Caputo, Eduardo Lacaz, Paulo R. Criado, Carlos M. Filho, Gilles Landman

**Affiliations:** ^1^ Dermatology Service of Faculdade de Medicina do ABC ABC University of Medicine Santo André Brazil

## 1 INTRODUCTION

Necrobiosis lipoidica (NL) and cutaneous sarcoidosis are granulomatous skin disorders of unknown etiology.^1^ Although these two diseases are distinct, they share histopathological and clinical features and, occasionally, one may mimic the other.^2^ Only a few cases of concomitant NL and sarcoidosis have been reported.^3^ Sarcoidosis is characterized by the formation of epithelioid and giant cell granulomas without caseous necrosis, and it may affect not only the skin but also many organs, most frequently (90%‐95% of cases) affecting the lungs.^4^, ^5^ Classic cutaneous lesions are erythematous papules, which become confluent forming a painless infiltrated plaque.^6^ These lesions may be seen in any part of the body, especially on the face, superior chest and extensor face of the limbs.^7^ In this report, a female patient, non‐diabetic, who was followed up along 20 years, presented different features of cutaneous sarcoidosis with no evidence of systemic invovement.

## 2 REPORT

**FIGURE 1:**
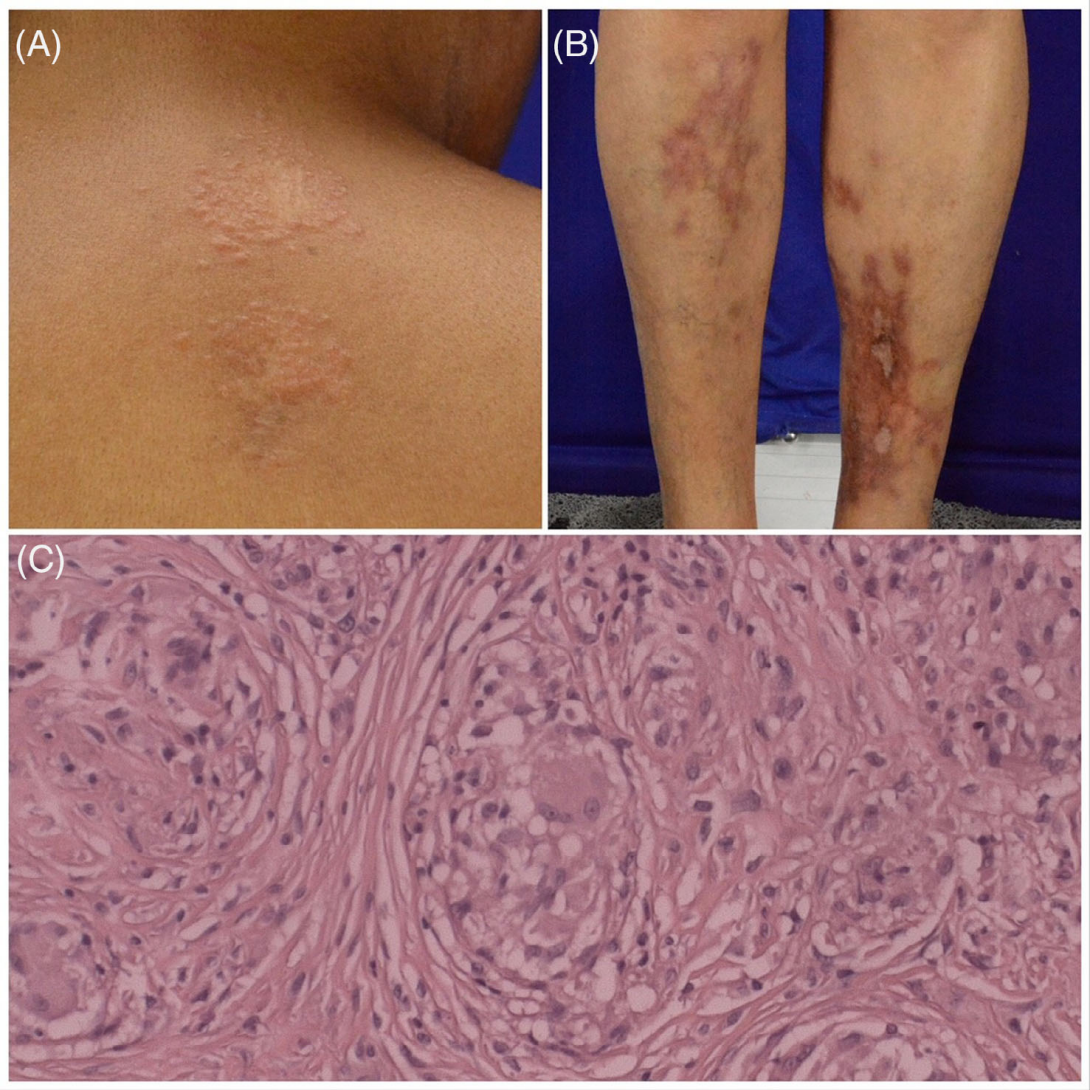
A, Yellow‐to‐orange papules become confluent to an infiltrated plaque on upper back; B, an atrophic reddish‐brown plaque with telangiectasias on the pretibial of the left lower limb; C, histopathology characterized by a granulomatous reaction with epithelioids histiocytes and multinucleated giant cells without surrounding lymphocytes

A non‐diabetic 44‐year‐old woman was referred to our dermatology center with a history of yellow‐to‐orange papules becoming confluent into an infiltrated plaque, initially, on the face, neck and upper back (Figure 1A). The rest of her skin and lymph nodes examination were unremarkable. Incisional biopsies from each of the eruptions were taken. The histology demonstrated epithelioid sarcoidal granulomas, negative acid‐alcohol–resistant bacilli with no fungus evidence. Further investigation was carried out showing negative tuberculosis screening, chest radiography with no abnormalities, absence of diabetes or insulin resistance, normal serum calcium, hepatic and renal functions preserved. The angiotensin‐converting enzyme dosage was normal. Due to a diagnosis of cutaneous sarcoidosis, a treatment was initiated. The patient started treatment with prednisone 20 mg, once daily, for 3 weeks. As there was no clinical response, the treatment was changed to intralesional injection (triamcinolone acetonide), one session every 15 days with a satisfactory response. Twenty years after her first clinical presentation, an atrophic reddish‐brown plaque with telangiectasias, similar to NL, appeared on the left lower limb (Figure 1B). Another incisional biopsy showed, on superficial and deep dermis, a granulomatous reaction pattern characterized by epithelioids histiocytes, multinucleated giant cells without surrounding lymphocytes (Figure 1C). Xanthomatous cells and plasma cells were absent. This finding fits most closely to a histological diagnosis of sarcoidosis. First, the patient was treated with clobetasol 0.05% ointment for 4 weeks with no clinical response. In addition, she was treated with intralesional injection of triamcinolone every 15 days. Lesions were significantly improved only after three sessions. Recurrent plaques were observed during her follow‐up.

## 3 DISCUSSION

The coexistence of cutaneous sarcoidosis and NL is rare.^8^ Lee et al^9^ described seven cases of patients with sarcoidosis who developed skin lesions similar to NL, however, histologically diagnosed as sarcoidosis. The close relation of these two diseases reinforce the question of whether they fall on the same spectrum of granulomatous skin disease or possibly share a final common pathway.^10^ Granulomatous disorders – including sarcoidosis, NL and granuloma annulare – show high concentrations of glioma‐associated oncogene homolog involved in cell signaling pathways similar to the embryonic development and the cell differentiation.^11^ In this report, the patient presented only cutaneous signals without systemic involvement in contrast to other cases reported in the literature whose major systemic signs have been associated with.^9^, ^12^ Cutaneous sarcoidosis mimicking NL appears to be difficult to treat in accordane with the literature.^9^ The histological findings that corroborate the diagnosis of sarcoidosis are: various clusters of epithelioid cells forming granulomas, scarcity of lymphocytes, presence of multinucleated giant cells and exclusion of an infectious cause or foreign body.^13^ The absence of mucin deposit and collagen degeneration rule out the diagnosis of NL.

## 4 CONCLUSIONS

With this report, we aim to demonstrate that there is a shared common pathogenic inflammatory and physicians shall consider sarcoidosis a differential diagnosis of NL.

## CONFLICT OF INTEREST

The authors have no conflict of interest to disclose.

## AUTHOR CONTRIBUTIONS

Conceptualization: Eduardo Lacaz, Paulo R. Criado

Resources: Gilles Landman, Carlos M. Filho

Supervision: Fernanda Simoneti

Writing – Original Draft Preparation: Ingrid Campos, Gabriela Caputo

Writing – Review & Editing: Ingrid Campos

 All authors have read and approved the final version of the manuscript.

 Corresponding author (Fernanda Simoneti, MD) had full access to all of the data in this study and takes complete responsibility for the integrity of the data and the accuracy of the data analysis.

## FINANCIAL SUPPORT

There was no supporting source/financial while conducting this study.

The lead author (Fernanda Simoneti, MD) affirms that this manuscript is an honest, accurate, and transparent account of the study being reported; that no important aspects of the study have been omitted; and that any discrepancies from the study as planned (and, if relevant, registered) have been explained.

## Data Availability

Data sharing is not applicable to this article as no new data were created or analyzed in this clinical case report.
